# Meta‐Research on the Science of Lived Experience Engagement in Mental Health and Substance Use Health Research: A Scoping Review and Qualitative Synthesis

**DOI:** 10.1111/hex.70733

**Published:** 2026-06-30

**Authors:** Lisa D. Hawke, Abigail Amartey, Joshua Dunphy, Hajar Seiyad, Joshua Orson, Shelby McKee, Terri Rodak

**Affiliations:** ^1^ University of Toronto Toronto Ontario Canada; ^2^ Centre for Addiction and Mental Health Toronto Ontario Canada

**Keywords:** lived experience, mental health, meta‐research, patient engagement, qualitative synthesis, scoping review, substance use

## Abstract

**Background:**

Partnering with people with lived experience of the mental health or substance use spectrum and caregivers is a growing priority in research. There is an emerging meta‐research literature on the science of engagement, but this has not been synthesised.

**Objective:**

This scoping review synthesises the meta‐research on the science of lived experience and caregiver engagement in mental health and substance use health research.

**Method:**

We conducted systematic searches in PsycInfo (Ovid), MEDLINE (Ovid), CINAHL (EBSCO), and Web of Science. Articles were eligible if they presented original meta‐research on lived experience/caregiver engagement in mental health and substance use health research in the past 10 years. Titles and abstracts, then full texts, were reviewed by two independent reviewers. Data were extracted into a spreadsheet and synthesised qualitatively.

**Results:**

Fifty‐seven articles were included. The meta‐research has aimed to understand experiences or needs in engagement contexts, describe or evaluate engagement groups, or develop or evaluate tools and resources to support engagement. The literature reports on positive experiences and challenges, describes approaches and new tools, and highlights opportunities for increased engagement. Papers point to the importance of improving engagement processes, structural supports and logistics.

**Conclusions:**

A growing body of meta‐research on the science of lived experience and caregiver engagement in mental health and substance use health research remains largely descriptive in nature, rather than analytical. Research teams are encouraged to seek solutions to identified challenges and key areas of development in engagement processes, structural supports and logistics. Addressing these aspects will further advance the meta‐research evidence base.

**Lived Experience:**

This review was conducted together with people with lived experience, including co‐authorship.

## 1 Background

Partnering with people with lived and living experience across the mental health and substance use health spectrum, and their caregivers, is a growing priority in the mental health and substance use health research sector [1]. Engagement takes the research out of the lab and puts it in the real world, focusing on the people most affected. Engagement can be implemented throughout the research process, from the initial idea generation stage to the mobilisation of the research findings [2, 3, 4]. Lived/living experience and caregiver engagement is particularly important in the mental health and substance use health research sector, due to issues such as the dominant role of subjective patient experiences as opposed to biomarkers [5]; the pressing need for epistemic justice [6, 7]; substantial power imbalances [8, 9]; and a marked history of marginalisation, coercive care, and direct harm to patients [10, 11]. Lived/living experience and caregiver engagement is guided by a pragmatic approach, where the everyday concerns and experiences of service users are used to directly inform the research [12].

A range of literature has discussed the impact of lived/living experience and caregiver engagement in mental health and substance use health research. Literature reviews have concluded that engagement positively affects multiple aspects of the research context, from the research environment and the components of the research project itself to the people engaged, the researchers, and the research participants [13, 14]. However, a previous scoping review found that most literature focuses primarily on subjective accounts of impact by the authors (e.g., reflection/description papers, qualitative studies, commentaries, etc.), rather than relying on objective, quantitative research [13].

Conducting research on the process of research itself, including its methodologies, is called ‘meta‐research’ [15]. Meta‐research helps improve research methodologies, approaches and scientific processes. This type of research on research ultimately aims to improve the state of evidence base as improved methodologies are implemented.

There is an emerging meta‐research literature on the science of lived/living experience and caregiver engagement in mental health and substance use health research. This includes commentary papers and literature reviews, as well as a broad range of original research in which the perspectives of lived/living experience and caregiver advisors are sought in a more formalised way than through usual engagement approaches, involving them as research participants [13, 16, 17, 18, 19]. Indeed, as the meta‐research on the science of engagement advances, publications on the topic are proliferating. However, despite multiple literature synthesis papers on the use and implementation of lived/living experience engagement [13, 20, 21, 22], to our knowledge, there is not yet a review available synthesising the meta‐research on the science of lived/living experience and caregiver engagement in the mental health and substance use health sector. A synthesis of the existing literature has the potential to guide research teams by highlighting documented processes, methodologies, challenges and gaps, which in turn can improve the meta‐research evidence base on the science of lived/living experience and caregiver engagement in mental health and substance use health research. This scoping review synthesises the original meta‐research on the science of lived/living experience and caregiver engagement in mental health and substance use health research.

## 2 Methods

The scoping review methodology was selected for this review based on the broad and exploratory research question [23]. A scoping review has five key steps: (1) defining the research question, (2) identifying relevant studies, (3) screening and selecting studies, (4) extracting data, and (5) summarising and reporting. To report on the review, we used the PRISMA Extension for Scoping Reviews (PRISMA‐ScR; Appendix A) [24]. We registered the protocol on Open Science Framework. Two lived/living experience advisors were engaged in the review, which we report on in accordance with the latest guidelines for reporting on engagement [25].

Our research question focuses on describing the meta‐research conducted on the science of lived/living experience and caregiver engagement in mental health and substance use health research. Following the PCC (population–concept–context) framework, the population of interest is people with lived/living experience of a mental health or substance use health condition, or caregivers, who are engaged in research. The concept is any aspect of the science of engagement derived from original research on the topic, that is, primary research studying lived/living experience and caregiver engagement in mental health and/or substance use health research. The context is academic meta‐research conducted anywhere in the world.

A health sciences research librarian (T.R.) developed, validated and finalised the search strategy in PsycInfo (Ovid). She provided a recommended list of databases relevant to our research question and subsequently translated the search strategy for additional databases as selected in collaboration with the research team. She ran the searches on October 23, 2025, in PsycInfo (Ovid), MEDLINE (Ovid), CINAHL (EBSCO), and Web of Science (Clarivate). The search strategies used database‐specific subject headings and keywords using natural language, combined with Boolean and advanced search operators to query two main concepts: lived/living experience and caregiver engagement in research AND mental health and/or substance use health. We limited the publications search to January 1, 2016, until October 23, 2025, with the goal of identifying recent meta‐research. We did not place any limitations on the type of publication or the language of publication within the search process. We removed review publications directly within the search strategy to eliminate the large number of reviews that were not relevant to the research question. Since we were interested in the meta‐research published in the academic literature, we did not conduct a grey literature search beyond dissertations and conference proceedings indexed in the selected databases. For the full PsycInfo search strategy, see Appendix B.

Table 1 presents the inclusion and exclusion criteria. Study screening and selection were performed in Covidence [26]. To be included, records had to reflect original meta‐research in the mental health and/or substance use health sector in which people with lived/living experience and caregivers with research engagement experience were participants. Lived/living experience and caregivers were defined according to the criteria and operationalisation of each included study. Language was limited to English and French based on the team's language skills. The past 10 years were retained as a study period in order to capture recent findings about engagement. Exclusions were records that did not represent original meta‐research on research engagement, that focused on engagement outside of the research engagement framework, that had participants who were not lived experience or caregiver advisors, or were in a language other than English and French. Discrepancies during full‐text screening were resolved through discussion, and consensus was reached between two reviewers (the lead investigator and a research analyst).

**Table 1:** Inclusion and exclusion criteria.

Inclusion criteria	Exclusion criteria
Original research	Commentaries, position papers, reviews or other publications that are not original research
Mental health and substance use health research	Engagement in clinical services, service design or other non‐research endeavour
Conducted on lived/living experience and caregiver engagement, as research participants	People with lived/living experience supported the research but did not participate in it
The report is in English and French	Outside of the language or time period scope
Last 10 years (2016–2025)	

Basic descriptive data were extracted by one team member into a spreadsheet and confirmed by a second team member (article reference, country of publication, year of publication, objective, method, population, sample size, mental health vs. substance use vs. concurrent disorder). We then synthesised the objectives, results, and future research needs qualitatively using NVivo qualitative data analysis software and a conventional content analysis approach [27]. Initial categories were deductively informed by familiarity with the literature following full‐text screening, followed by inductive and iterative coding. This involved a data analyst extracting and coding relevant article text, with initial categories refined and additional categories developed that reflected emerging insights and patterns in the data. Themes and sub‐themes were subsequently finalised following discussion between the lead investigator and data analyst. Results are summarised in narrative and table format.

### 2.1 Lived/Living Experience Engagement

Engagement is reported in Table 2 following recent reporting guidelines for lived/living experience and caregiver engagement in mental health and substance use health research [25].

**Table 2:** Research engagement of Lived/Living Experience and Caregiver Working Group.

Domain	Description
1. Who was involved?	The team included two people with lived/living experience as project advisors. Both advisors had previous research engagement experience, including experience advising on scoping review studies.
2. What were the activities, roles and responsibilities?	We held one engagement consultation meeting when designing the study to discuss the research questions, concept and database terms. We then held a meeting to discuss the specific research questions, the article set, and the data to be extracted from the articles, revising our data extraction variables together. Upon completion of a draft of the results section, we met again to review the results, solicit feedback and brainstorm on the discussion section together. They then reviewed the draft version of the current manuscript in their own time and provided feedback to the team in written format. The lived/living experience advisors are co‐authors on the manuscript.
3. How did you go about the engagement process?	There was a light‐to‐moderate engagement level in this modest project. We held consultation meetings with the advisors, accompanied by asynchronous work and co‐authorship. Decisions regarding all aspects of the project were reached by consensus between the lead investigator, one research staff member and the two advisors. Our engagement activities were held virtually. Advisors were paid for their contributions at an hourly rate.
4. When did engagement occur?	We conducted engagement from the initial conceptualisation of the project to the finalisation of the manuscript. Three meetings and asynchronous work were sufficient to support this modest project.
5. What was the impact?	By engaging advisors with lived/living experience and with experience in an engagement capacity, we ensured that this review was relevant to real‐world experiences of being engaged in mental health and substance use health research. The advisors provided important feedback that helped form the manuscript direction. Our experience working together with people with lived/living experience confirms the value of their contributions to scoping reviews.

## 3 Results

**Figure 1:**
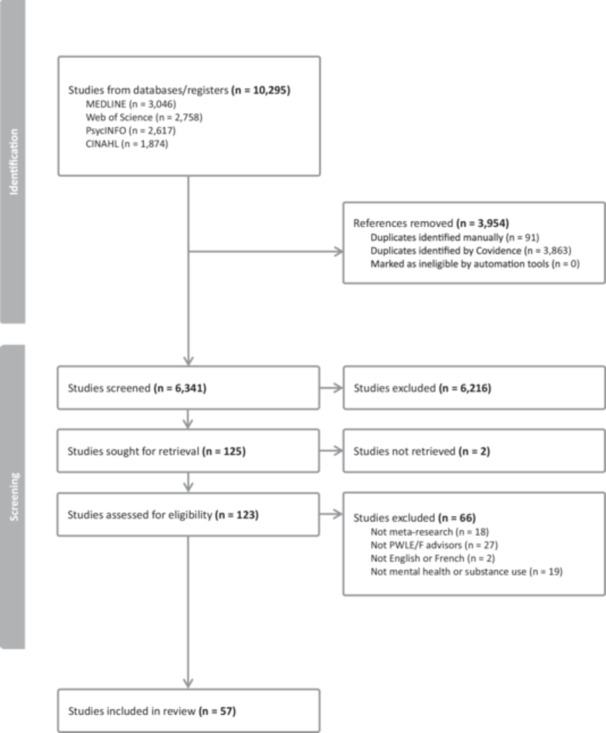
PRISMA flow chart for documents identified in the scoping review.

A total of 57 publications were retained in the review (for the PRISMA diagram, see Figure 1). Articles are summarised in Table 3. There has been a progressive increase in the number of meta‐research articles emerging over the past 10 years, doubling in the past 2 years. The majority of papers emerged from the United Kingdom, Canada and Australia. Most were qualitative in nature (77.19%), with a few mixed‐methods approaches and limited quantitative (Delphi). Sample sizes were mostly small (81% of reported sample sizes included < 21 participants, including nearly one‐half of studies with < 11 participants). Youth or older‐adult‐oriented papers were a minority, as were substance use‐related papers. About a quarter of the papers included caregivers. In terms of models of engagement followed, 29 (50.9%) studies did not point to any model or framework. The remaining articles referred to a wide range of models and guidance frameworks, with no consensus (a total of 17 unique models or frameworks). Eight (14.0%) referred to community‐based participatory research or participatory action research as general research engagement approaches, without a specific engagement model or framework cited. Detailed findings are reported in Table 4.

**Table 3:** Descriptive summary of articles included in the review.

Article characteristics		*n* (%)
Year of publication	2016–2017	4 (7.02%)
	2018–2019	5 (8.77%)
	2020–2021	12 (21.05%)
	2022–2023	12 (21.05%)
	2024–2025	24 (42.11%)
Country of publication	The United Kingdom	18 (31.58%)
	Canada	14 (24.56%)
	Australia	12 (21.05%)
	The United States	7 (12.28%)
	Norway	2 (3.51%)
	The Netherlands	1 (1.75%)
	Austria	1 (1.75%)
	Sweden	1 (1.75%)
	Spain	1 (1.75%)
Method	Qualitative	44 (77.19%)
	Mixed‐method	13 (22.81%)
Population age	Child‐specific papers	0 (0%)
	Youth‐specific papers	12 (21.05%)
	Adult or general papers	43 (75.44%)
	Older adult‐specific papers	2 (3.51%)
Sample size	1–10	25 (43.86%)
	11–20	17 (29.82%)
	21–30	7 (12.28%)
	31+	3 (5.26%)
	Not reported	5 (8.77%)
Included caregivers	Yes	14 (24.56%)
	No	43 (75.44%)
Condition(s)	Mental health	42 (73.68%)
	Substance use health	8 (14.04%)
	Mental health and substance use health	7 (12.28%)
	Concurrent mental health and substance use health	0 (0.0%)

**Table 4:** Themes generated from a conventional content analysis of the included studies, with the number and percentage of papers and corresponding references.

Category	Theme	*n* (%)	References
Objectives	Understand experiences or needs in engagement	32 (56.1%)	[8, 25, 28, 29, 30, 31, 32, 33, 34, 35, 36, 37, 38, 39, 40, 41, 42, 43, 44, 45, 46, 47, 48, 49, 50, 51, 52, 53, 54, 55, 56, 57]
	Describe or evaluate engagement groups	24 (42.1%)	[9, 18, 34, 58, 59, 60, 61, 62, 63, 64, 65, 66, 67, 68, 69, 70, 71, 72, 73, 74, 75, 76, 77, 78]
	Develop or evaluate engagement guidelines, frameworks or tools	5 (8.8%)	[25, 44, 66, 79, 80]
Results	Reported positive engagement experiences and impacts	48 (84.2%)	[8, 9, 18, 28, 30, 31, 32, 33, 34, 35, 38, 39, 41, 42, 44, 45, 46, 47, 48, 49, 50, 51, 52, 53, 54, 55, 56, 57, 58, 59, 60, 61, 62, 63, 64, 65, 66, 68, 69, 71, 72, 73, 74, 75, 76, 77, 78, 80]
	Described challenges in engagement	41 (71.9%)	[8, 9, 28, 29, 32, 34, 35, 36, 38, 39, 40, 41, 42, 43, 44, 45, 46, 47, 48, 49, 51, 52, 53, 55, 56, 58, 60, 61, 62, 63, 64, 65, 67, 69, 70, 72, 73, 74, 75, 76, 77]
	Described engagement procedures or approaches	19 (33.3%)	[18, 25, 30, 31, 38, 40, 49, 54, 56, 57, 60, 64, 65, 66, 67, 68, 70, 74, 75]
	Highlighted opportunities for increased engagement	15 (26.3%)	[18, 30, 31, 33, 37, 48, 50, 55, 57, 58, 62, 64, 72, 77, 80]
	Developed, evaluated or identified a need for guidelines, frameworks or tools	7 (12.3%)	[25, 44, 66, 70, 75, 79, 80]
Future developments	Improve engagement processes	36 (63.2%)	[8, 25, 28, 29, 33, 34, 35, 37, 38, 41, 42, 44, 47, 49, 50, 51, 53, 55, 57, 58, 61, 63, 64, 65, 66, 68, 71, 72, 73, 74, 75, 76, 78, 79, 80]
	Improve structural supports for engagement	14 (24.6%)	[18, 25, 30, 32, 35, 42, 43, 50, 55, 57, 60, 62, 65, 73]
	Improve logistical aspects of engagement	4 (7.0%)	[33, 35, 36, 58]

Through the conventional content analysis of the papers, themes were generated across three categories of findings: objectives, results and future research recommendations. For a description of the themes and reference to the associated papers, see Table 3.

### 3.1 Objectives of Included Studies

#### 3.1.1 Understand Experiences or Needs in Engagement

In terms of the objectives of the included studies, over half aimed to understand the experiences and needs of individuals in the engagement setting [8, 25, 28, 29, 30, 31, 32, 33, 34, 35, 36, 37, 38, 39, 40, 41, 42, 43, 44, 45, 46, 47, 48, 49, 50, 51, 52, 53, 54, 55, 56, 57]. For example, many included aims to understand the concrete engagement experiences of lived experience advisors and/or researchers [8, 28, 31, 32, 34, 35, 38, 43, 44, 45, 48, 50, 51, 52, 53, 56, 57] or overarching perspectives on engagement [40, 41, 42, 46, 49, 54, 55]. A few articles described objectives around stimulating reflection on their experience of the collaborative partnerships [29, 30, 35, 39].

#### 3.1.2 Describe or Evaluate Engagement Groups

A large proportion of the literature also aimed to describe and/or evaluate an engagement group [9, 34, 58, 59, 60, 61, 62, 63, 64, 65, 66, 67, 68, 69, 70, 71, 72, 73, 74, 75, 76, 77, 78]. This included a number of articles presenting case studies of an engagement group supporting a specific project or organisation [9, 18, 59, 61, 62, 63, 70, 72, 73, 74, 76]. Some aimed to describe the process of building, implementing or operationalising an engagement group [18, 62, 63, 70, 72]. Others reported aims to describe or evaluate the methods used to engage or the contributions that advisors made to the project [34, 58, 60, 64, 65, 67, 68].

#### 3.1.3 Develop or Evaluate Engagement Guidelines, Frameworks or Tools

A few articles explicitly aimed to develop or evaluate guidelines, frameworks or tools to support the engagement process [25, 44, 66, 79, 80]. Several aimed to develop frameworks to support the use of authentic engagement practices, broadly speaking [44, 66, 79, 80]. One study aimed to develop guidelines to support the reflexive reporting of the engagement process [25].

### 3.2 Results of Included Studies

#### 3.2.1 Reported Positive Engagement Experiences and Impacts

The results of the meta‐research are varied across studies. A large proportion of the literature presented results that described positive engagement experiences and impacts [8, 9, 18, 28, 30, 31, 32, 33, 34, 35, 38, 39, 41, 42, 44, 45, 46, 47, 48, 49, 50, 51, 52, 53, 54, 55, 56, 57, 58, 59, 60, 61, 62, 63, 64, 65, 66, 68, 69, 71, 72, 73, 74, 75, 76, 77, 78, 80]. These included positive engagement experiences in the case described [8, 9, 18, 28, 30, 31, 32, 33, 34, 39, 41, 44, 45, 46, 50, 51, 53, 54, 55, 56, 57, 58, 61, 62, 65, 68, 72, 73, 76, 77, 78, 80]. There were also many positive personal impacts of the engagement experience [28, 32, 33, 35, 38, 41, 45, 48, 49, 52, 53, 55, 56, 57, 62, 64, 68, 69, 73, 75, 76, 77, 78], such as feeling heard, valued, accepted and destigmatised while gaining valuable opportunities for learning, social connection, meaning, empowerment, fulfilment and enjoyment. Other personal impacts include increased self‐confidence, a sense of purpose, advancement in recovery, and personal growth. Broader positive impacts were also described, such as improved research success, a better research culture, and greater responsiveness to and buy‐in by the local community [8, 39, 42, 47, 49, 50, 53, 57, 63, 64, 66, 69, 71, 72, 73, 74, 75].

#### 3.2.2 Described Challenges in Engagement

Many papers presented results sections that catalogued some of the challenges encountered in lived experience engagement contexts [8, 9, 28, 29, 32, 34, 35, 36, 38, 39, 40, 41, 42, 43, 44, 45, 46, 47, 48, 49, 51, 52, 53, 55, 56, 58, 60, 61, 62, 63, 64, 65, 67, 69, 70, 72, 73, 74, 75, 76, 77]. These were varied, including challenges with recruitment and retention (including diversity gaps) [28, 34, 36, 40, 44, 45, 47, 53, 55, 58, 62, 73, 77]. Various challenges were encountered in terms of the process of conducting authentic engagement [29, 34, 35, 41, 46, 47, 51, 56, 60, 63, 67, 69, 70, 72, 74, 75, 76]. A number of challenges were identified at the level of the researcher and engagement practitioner, including relational challenges [32, 36, 41, 42, 44, 49, 61, 67, 73]. For some, challenges were encountered in navigating lived experience identities in the research space [9, 36, 38, 40, 41, 42, 51, 60, 62, 75], which was combined with negative personal impacts [36, 38, 42, 48, 51, 55, 60]. Challenges were further encountered in terms of managing the concrete logistics of engagement [39, 43, 44, 48, 52, 53, 55, 65, 72, 73, 76]. Lastly, numerous challenges were reported at the structural or institutional level, such as navigating payment processes, institutional rules and regulations, and systemic and power‐related pressures [8, 9, 43, 51, 63, 70, 74].

#### 3.2.3 Described Engagement Procedures or Approaches

In a third of the papers, the results included descriptions of the engagement procedures or approaches undertaken to facilitate positive engagement [18, 25, 30, 31, 38, 40, 49, 54, 56, 57, 60, 64, 65, 66, 67, 68, 70, 74, 75]. These included aspects such as providing individual and managerial support [18, 31, 56, 57, 64, 67], undertaking relationship‐building activities as part of the engagement process [30, 38, 40, 60, 68, 74], ensuring flexibility and accessibility [30, 31, 40, 49, 75], or providing advisor training [31, 66].

#### 3.2.4 Highlighted Opportunities for Increased Engagement

Over a quarter of the included studies highlighted opportunities for increased engagement [18, 30, 31, 33, 37, 48, 50, 55, 57, 58, 62, 64, 72, 77, 80]. For example, some noted that researchers and systems want and need to offer more opportunities for advisors to become engaged and that engagement can be enhanced across the project [30, 31, 50, 62, 64, 72]. It was noted that advisors were interested in accessing more engagement opportunities [30, 37, 48, 58, 77, 80]. This is included within individual projects and more broadly at the engagement systems level. More training, more contributions to analysis, more network development, and more uptake of engagement as a whole were all recommended.

#### 3.2.5 Developed, Evaluated or Identified a Need for Guidelines, Frameworks or Tools

A few included papers presented results around the development or evaluation of a guideline, framework or tool [25, 44, 66, 70, 75, 79, 80]. These notably included frameworks to support the process of lived/living experience engagement [44, 70, 79]. One presented results around the relevance of an engagement reporting guideline, alongside an accompanying guideline for use [25]. Two studies found that there was a need to develop such materials to better support those conducting engagement [25, 66].

### 3.3 Future Developments

#### 3.3.1 Improve Engagement Processes

The last category of analysis was recommendations for future research and operational improvements to guide the meta‐research on engagement. Many papers pointed to the need for improved engagement processes as a whole [8, 25, 28, 29, 33, 34, 35, 37, 38, 41, 42, 44, 47, 49, 50, 51, 53, 55, 57, 58, 61, 63, 64, 65, 66, 68, 71, 72, 73, 74, 75, 76, 78, 79, 80]. Notably, these included further evaluating engagement, such as evaluating how advisors contribute to research [37, 44, 63, 66, 72, 73, 76, 79], including in specific populations such as youth, students and people with dementia. Longitudinal evaluations were further recommended. Additional areas of process improvement included establishing a grievance system [35], increasing participation and diversity [25, 28, 34, 44, 47, 49, 53, 55, 56, 64, 79], and implementing and improving upon guidelines and frameworks that aim to improve engagement practices [25, 35, 44, 50, 51, 61, 79, 80]. In line with the results section, the articles also highlighted the need for a more comprehensive integration of engagement in future research [29, 33, 35, 37, 38, 41, 42, 44, 49, 65, 68, 71, 74].

#### 3.3.2 Improve Structural Supports for Engagement

Several articles suggested improving the structural supports for engagement as an area of future development [18, 25, 30, 32, 35, 42, 43, 50, 55, 57, 60, 62, 65, 73]. Authors pointed to the importance of improving funding for engagement in research as a prerequisite to all engagement development [30, 35, 42, 43, 50, 55, 57, 60, 62], alongside other broad structural supports. These structural supports, which would help amplify lived/living experience and caregiver voices, included the development of institutional and stakeholder policies that support researcher–advisor relationships [30], establishing frameworks, governance, oversight, and advisory boards at the institutional level [18, 35, 50, 65], greater support for training and skill development [32, 35, 73], increased understanding among research ethics board staff of considerations relevant to research engagement [35], and modifications to journal and publishing practices that facilitate engagement reporting [25].

#### 3.3.3 Improve Logistical Aspects of Engagement

Lastly, a few author groups called for improvements in logistical support [33, 35, 36, 58]. These included considerations for improved advisor training [35, 58], clarity around meeting structures that build trust [33], and the need for time and flexibility in research infrastructures to accommodate the flexibilities needed to conduct engagement authentically [36].

## 4 Discussion

This scoping review synthesised the meta‐research on the engagement of people with lived/living experience and caregivers in mental health and substance use health research. We found a moderate and growing body of meta‐research examining the science of engagement, although most of the literature aims to understand engagement experiences and describe specific engagement groups. Positive engagement experiences, challenges and approaches were described, alongside an overarching call for increased engagement activities. A few studies have attended to the development or evaluation of guidelines, tools or frameworks to advance the science of engagement, but these remain rare. The literature points to the importance of improving engagement processes, structural supports and logistics to advance the science of engagement in this research sector in the future.

It is important to note that the majority of the meta‐research on engagement in mental health and substance use health is largely descriptive, rather than analytical. We see the volume of meta‐research increasing in recent years, which mirrors the increase in the volume of research that includes engagement [13]. However, this reveals that the science of engagement remains at an early exploratory stage as the discipline strives to understand research engagement at its foundation. This early exploratory stage may further influence the aspects of research engagement being reported on. Only a limited number of studies explicitly called for improved logistical aspects of engagement, and while this suggests an area of underreporting, it could also reflect aspects that are embedded within structural and/or process recommendations. For example, logistical considerations like advisor compensation may fall under funding‐related structural needs, while improved meeting structure or flexibility may fit within broader discussions of how to improve engagement processes. This highlights a potential need for a clearer distinction between day‐to‐day logistical aspects and the structural and process needs that influence them.

Engagement remains a relatively new area of science, and it is now time for a new, emerging evidence base to attend to improving processes. This is reflected in the recent emergence of a few frameworks, guidelines and recommendations in terms of concrete tools [25, 44, 66, 80]. Research teams are encouraged to continue reflecting on the barriers and challenges encountered in engagement contexts and addressing those challenges with concrete tools, resources and solutions [81]. They are also encouraged to consider and report upon the degree to which the engagement is embedded in an organisational culture or not, which may affect the motivations for doing it, the way it is conducted and evaluated, as well as the impact it has on the work being done [82].

It is also apparent in the literature that certain groups have not yet been attended to rigorously in the meta‐research on engagement. Specifically, we did not find any meta‐research on engagement in lower‐middle‐income countries, despite emerging work describing lived/living experience engagement in the mental health sector in these contexts [83]. Likewise, we did not find any meta‐research on the engagement of children, which is an area where guidance would seem to be important, given the need for different approaches based on developmental level [84]. There was a limited amount of research focusing on older adults and caregivers, both of which may have specific considerations to take into account in engagement settings [85, 86]. Similarly, it is important to better understand potential differences in research engagement practices across diverse settings (i.e., inpatient, outpatient, community‐based settings, etc.) and research topics (e.g., coercive practices). Engagement in mental health and substance use health is often addressed separately, and concurrent disorders are not addressed at all, despite high degrees of overlap and comorbidity among individual experiences [87]. This reveals important gaps in the meta‐research that should be addressed by research teams going forward.

The majority of the meta‐research on engagement is qualitative in orientation. It is important to qualitatively explore the engagement of people with lived/living experience and caregivers in mental health and substance use health research, especially at this early stage of the meta‐research in which we are aiming to gain an in‐depth understanding of engagement experiences and develop new theories about engagement models [88]. However, there are emerging quantitative measures and innovative approaches to the measurement of engagement that should also be applied [89], making it possible to compare engagement experiences across the literature in quantitative or mixed‐methods manners. Meta‐researchers are encouraged to consider how quantitative approaches, and especially mixed‐methods approaches [90], might enhance the evidence on research engagement.

### 4.1 Strengths and Limitations

This review on engagement was conducted with engagement embedded throughout. Beyond descriptive reporting, we conducted a qualitative synthesis of a unique area of research. However, several limitations should be kept in mind when interpreting the results. Notably, we only reviewed the literature emerging in the past 10 years and might have therefore missed some early findings. We only searched English‐language databases and were only open to articles published in English and French, given the language capabilities of the team; all selected articles were published in English. We chose not to include non‐primary research studies or expand into the grey literature other than including student dissertations and conference abstracts, which may have limited the potential for additional resources on lived experience engagement research.

## 5 Conclusions

There is a rapidly growing body of meta‐research attending to the science of lived/living experience and caregiver engagement in mental health and substance use health research. However, the literature remains largely descriptive in nature, rather than analytical, and several important populations are under‐researched to date. Research teams are encouraged to seek concrete solutions to identified barriers or challenges and address key areas of development in engagement processes, structural supports and logistics. Addressing these aspects will continue to advance the evidence base on research engagement.

## Author Contributions

**Lisa D. Hawke:** conceptualisation, investigation, funding acquisition, writing – original draft, methodology, formal analysis, project administration, supervision, resources. **Abigail Amartey:** methodology, formal analysis, writing – review and editing, investigation, data curation. **Joshua Dunphy:** investigation, writing – review and editing, formal analysis, validation, data curation. **Hajar Seiyad:** investigation, writing – review and editing, methodology. **Joshua Orson:** investigation, methodology, writing – review and editing. **Shelby McKee:** investigation, formal analysis, validation, writing – review and editing, data curation. **Terri Rodak:** investigation, writing – review and editing, methodology, data curation.

## Ethics Statement

The authors have nothing to report.

## Consent

The authors have nothing to report.

## Conflicts of Interest

The authors declare no conflicts of interest.

## Permission to Reproduce Material From Other Sources

Not applicable.

## Clinical Trial Registration

Not applicable.

## Supporting information

Supporting File 1

Supporting File 2

## Data Availability

The data that support the findings of this study are available from the corresponding author upon reasonable request. No original data were collected as part of this study. The data relevant to the review are included herein.
