# Supplementary material for: Meta‐Research on the Science of Lived Experience Engagement in Mental Health and Substance Use Health Research: A Scoping Review and Qualitative Synthesis
Source: Health Expect. 2026 Jun 30;29(4):e70733. doi: 10.1111/hex.70733 (PMC13316132; doi:10.1111/hex.70733)
Supplement: Supplementary file 2 — Supporting File 2 [file HEX-29-e70733-s001.docx]

**Appendix B**

Search conducted on October 23, 2025

| **APA PsycInfo <1806 to October 2025 Week 2>** | | |
| --- | --- | --- |
| 1 | exp Mental Disorders/ | 1133845 |
| 2 | exp Psychiatry/ | 60980 |
| 3 | (mental* or psychiatr*).ti,id,hw. | 527415 |
| 4 | (mental* or psychiatr* or mood* or affective* or anxiety or depress* or dysthymi* or phobia* or panic* or obsess* or compuls* or OCD or bipolar or bi-polar or personality disorder* or borderline personalit* or manic or mania or psychosis or psychoses or psychotic or schizo* or delusion* or (hear* adj2 voice*)).ti,id,hw. or (mental* or psychiatr* or mood* or affective* or anxiety or depress* or dysthymi* or phobia* or panic* or obsess* or compuls* or OCD or bipolar or bi-polar or personality disorder* or borderline personalit* or manic or mania or psychosis or psychoses or psychotic or schizo* or delusion* or (hear* adj2 voice*)).ab. /freq=3 | 1097426 |
| 5 | (post-trauma* or posttrauma* or PTSD or complex trauma or developmental trauma or CPTSD).ti,id,hw. or (post-trauma* or posttrauma* or PTSD or complex trauma or developmental trauma or CPTSD).ab. /freq=3 | 58175 |
| 6 | ((disorder* adj2 eating) or anorex* or bulimi*).ti,id,hw. or ((disorder* adj2 eating) or anorex* or bulimi*).ab. /freq=3 | 40902 |
| 7 | (suicid* or selfharm* or self-harm* or selfinjur* or self-injur*).ti,id,hw. or (suicida* or selfharm* or self-harm* or selfinjur* or self-injur*).ab. /freq=3 | 68578 |
| 8 | ((behavio?r* or gambl* or shop* or buy* or spend* or steal* or theft*) adj3 (disorder* or addiction* or compuls* or problem* or pathological*)).ti,id,hw. or ((behavio?r* or gambl* or shop* or buy* or spend* or steal* or theft*) adj3 (disorder* or addiction* or compuls* or problem* or pathological*)).ab. /freq=3 | 70890 |
| 9 | (Kleptomania* or Trichotillomania or (hair adj3 pull*) or dermatillomania or excoriation or (skin adj3 pick*)).ti,id,hw. or (Kleptomania* or Trichotillomania or (hair adj3 pull*) or dermatillomania or excoriation or (skin adj3 pick*)).ab. /freq=3 | 1708 |
| 10 | (neurodiver* or neuro-diver* or attention deficit* or ADHD or autism or autistic or asperger* or ASD or tourette*).ti,id,hw. or (neurodiver* or neuro-diver* or attention deficit* or ADHD or autism or autistic or asperger* or ASD or tourette*).ab. /freq=3 | 107222 |
| 11 | Emotion* dysregulation.ti,id,hw. or Emotion* dysregulation.ab. /freq=3 | 2649 |
| 12 | Oppositional defian*.ti,ab,id,hw. | 4333 |
| 13 | (Conduct adj3 (disorder* or problem* or issue*)).ti,id,hw. or (Conduct adj3 (disorder* or problem* or issue*)).ab. /freq=3 | 7240 |
| 14 | (Behavio?r* adj3 (disorder* or problem* or disrupt*)).ti,id,hw. or (Behavio?r* adj3 (disorder* or problem* or disrupt*)).ab. /freq=3 | 62093 |
| 15 | (externaliz* or externalis* or internalize* or internalis*).ti,id,hw. or (externaliz* or externalis* or internalize* or internalis*).ab. /freq=3 | 14431 |
| 16 | (dementia* or alzheimer*).ti,id,hw. or (dementia* or alzheimer*).ab. /freq=3 | 110351 |
| 17 | ((substance* or drug* or tobacco or nicotine or alcohol* or cannabis or marijuana or stimulant* or steroid* or analgesic or sedative* or cocaine or hallucinogen* or psilocybin or amphetamine* or methamphetamine* or benzodiazepine* or opioid* or opiate* or heroin or fentanyl or inhalant* or depressant* or barbiturate*) adj3 ("use" or user* or misus* or abus* or disorder* or depend* or addict* or withdraw* or detox* or overdose* or recovery)).ti,id,hw. or ((substance* or drug* or tobacco or nicotine or alcohol* or cannabis or marijuana or stimulant* or steroid* or analgesic or sedative* or cocaine or hallucinogen* or psilocybin or amphetamine* or methamphetamine* or benzodiazepine* or opioid* or opiate* or heroin or fentanyl or inhalant* or depressant* or barbiturate*) adj3 ("use" or user* or misus* or abus* or disorder* or depend* or addict* or withdraw* or detox* or overdose* or recovery)).ab. /freq=3 | 211291 |
| 18 | SUD.ti,ab,id,hw. | 6198 |
| 19 | ("People who use drugs" or PWUD).ti,ab,id,hw. | 1357 |
| 20 | ((assist* or supervis* or manag* or treat* or inpatient* or residential*) adj3 (withdraw* or detox*)).ti,ab,id,hw. | 5004 |
| 21 | ((opioid* or opiate*) adj3 (replacement or substitution or maintenance)).ti,ab,id,hw. | 1872 |
| 22 | (opioid agonist* adj3 (treatment* or therap*)).ti,ab,id,hw. | 1023 |
| 23 | ((medica* assisted or opioid assisted) adj3 (treatment* or therap*)).ti,id,hw. or ((medica* assisted or opioid assisted) adj3 (treatment* or therap*)).ab. /freq=3 | 665 |
| 24 | (naloxone or methodone or buprenorphine or suboxone or sublocade).ti,id,hw. or (naloxone or methodone or buprenorphine or suboxone or sublocade).ab. /freq=3 | 7021 |
| 25 | harm reduction.ti,id. or harm reduction.ab. /freq=2 | 4625 |
| 26 | or/1-25 | 1717681 |
| 27 | client participation/ | 3760 |
| 28 | (co-produc* or coproduc*).ti,id. | 942 |
| 29 | (co-design* or codesign*).ti,id. | 809 |
| 30 | (co-creat* or cocreat*).ti,id. | 1852 |
| 31 | (co-construct or coconstruct*).ti,id. | 101 |
| 32 | (collaborat* adj2 methodol*).ti,id. | 31 |
| 33 | ("patient* and public* involvement" or "public* and patient* involvement").ti,id. | 351 |
| 34 | advisory group*.ti,id. | 68 |
| 35 | expert* by experience*.ti,id. | 132 |
| 36 | community expert*.ti,id. | 8 |
| 37 | peer researcher*.ti,ab,id. | 183 |
| 38 | peer educator*.ti,id. | 171 |
| 39 | participatory action research.ti,ab,id. | 3315 |
| 40 | ((patient* or client* or "service user*" or consumer* or survivor* or informant* or family or families or carer* or caregiver* or communit* or peer* or child* or youth or young people) adj3 (participat* or engag* or advisor* or partner* or involv* or collaborat* or consult*) adj5 research*).ti,id. | 3238 |
| 41 | (partner* adj2 (participat* or engag* or advisor* or involv* or collaborat* or consult*) adj5 research*).ti,id. | 83 |
| 42 | ((patient* or client* or "service user*" or consumer* or survivor* or informant* or family or families or carer* or caregiver* or communit* or peer* or child* or youth or young people) adj3 (participat* or engag* or advisor* or partner* or involv* or collaborat* or consult*)).ti,id. and research.id. | 3807 |
| 43 | ((patient* or client* or "service user*" or consumer* or survivor* or informant* or family or families or carer* or caregiver* or communit* or peer* or child* or youth or young people) adj3 (participat* or engag* or advisor* or partner* or involv* or collaborat* or consult*)).ti,id. and research.ab. /freq=3 | 3313 |
| 44 | (("lived expertise" or "living expertise" or "lived experience" or "living experience" or PWLE* or "people living with") adj5 (participat* or engag* or advisor* or partner* or involv* or collaborat* or consult* or lead or led or researcher* or voice*)).ti,id. | 300 |
| 45 | ((user*-centred or user*-centered or user* oriented or patient*-centred or patient*-centered or patient* oriented or client-centered or client-centred or client* oriented or experience-based) adj3 (partnership* or research* or collaborat*)).ti,id. | 280 |
| 46 | ((collaborat* or participatory) adj2 research*).ti,id. and (patient* or client* or child* or youth or young people* or "service user*" or consumer* or survivor* or partner* or informant* or communit* or peer* or family or families or carer* or caregiver* or "lived expertise" or "living expertise" or "lived experience" or "living experience" or PWLE* or "people living with").ti,id,hw. | 3552 |
| 47 | or/27-46 | 16863 |
| 48 | 26 and 47 | 4045 |
| 49 | limit 48 to yr="2016 -Current" | 2743 |
| 50 | (systematic review or scoping review or realist review or rapid review or narrative review or umbrella review or metaanalysis or meta-analysis).ti,id. | 81113 |
| 51 | 49 not 50 | 2617 |
